# Supplementary material for: I Choose You: Selecting Accurate Reference Genes for qPCR Expression Analysis in Reproductive Tissues in Arabidopsis thaliana
Source: Biomolecules. 2023 Mar 2;13(3):463. doi: 10.3390/biom13030463 (PMC10046263; doi:10.3390/biom13030463)
Supplement: Supplementary file 1 [file biomolecules-13-00463-s001.zip › biomolecules-2188067-Supplemental Information.pdf]

Supplemental Information

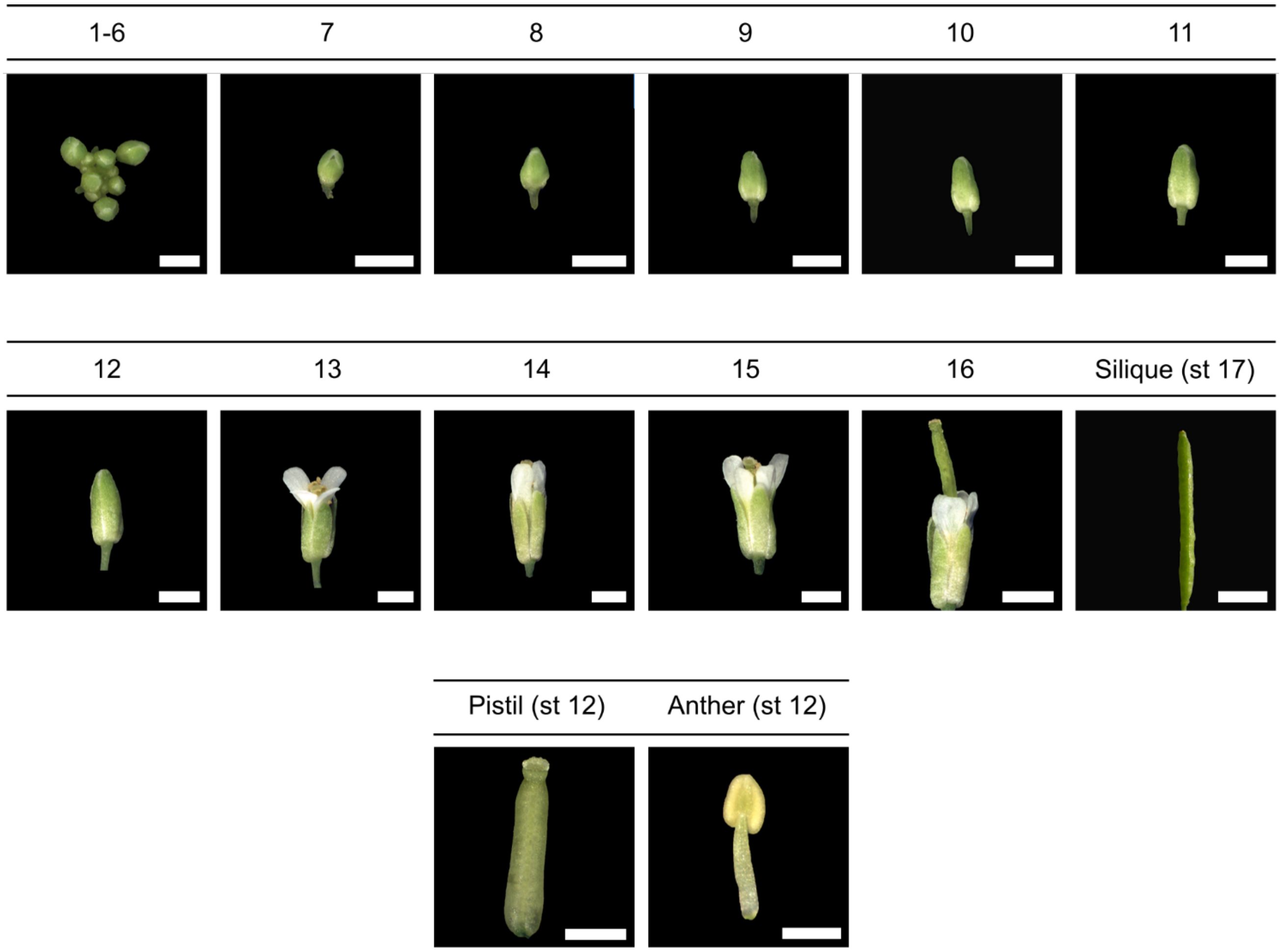

Figure S1. Stages of flower development and tissues of *A. thaliana* used in this study. Images representing the developmental stages and tissues of *A. thaliana* flowers according to [38]. All images were obtained with a stereo microscope. Scale bars: 5 mm [Silique (st 17)], 1 mm (st 1-16) and 0.5 mm [Anther (st 12) and Pistil (st 12)]. Abbreviations: st, stage.

UBC9

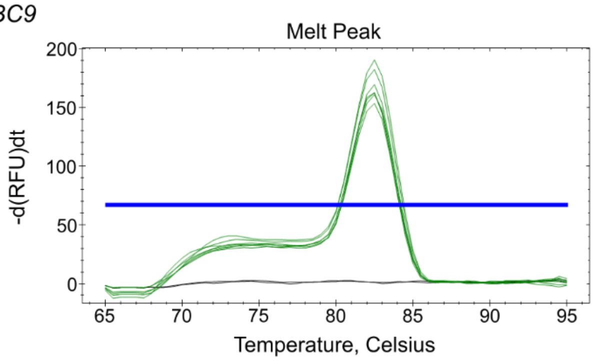

ACT7

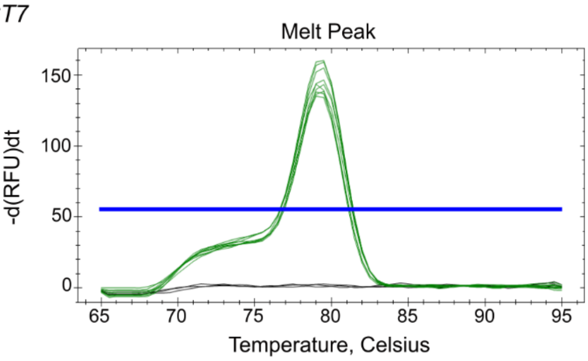

GAPC-2

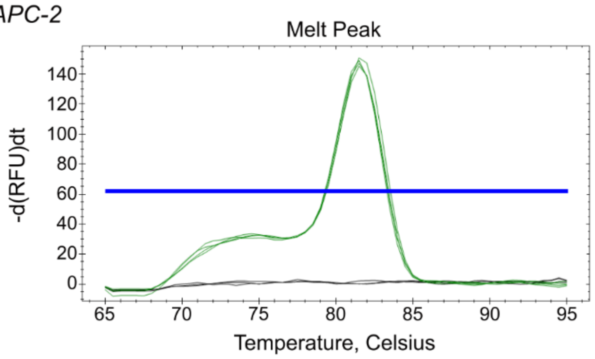

RCE1

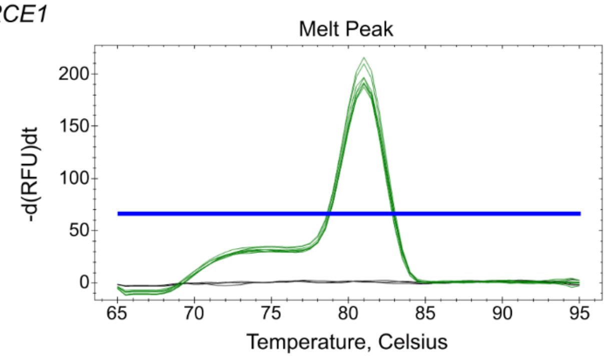

PP2AA3

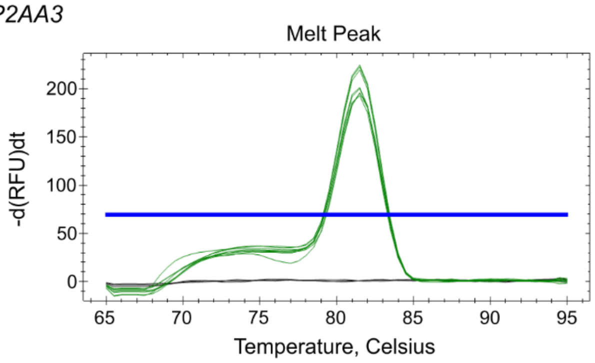

TUA2

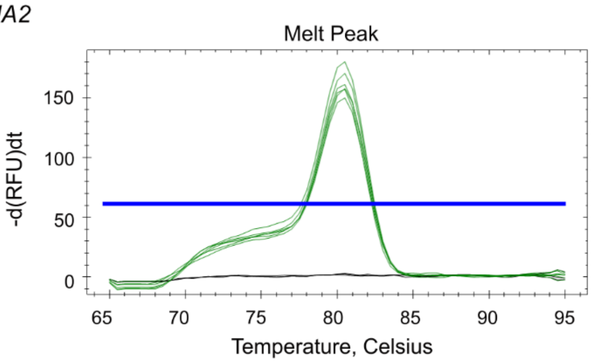

SAC52

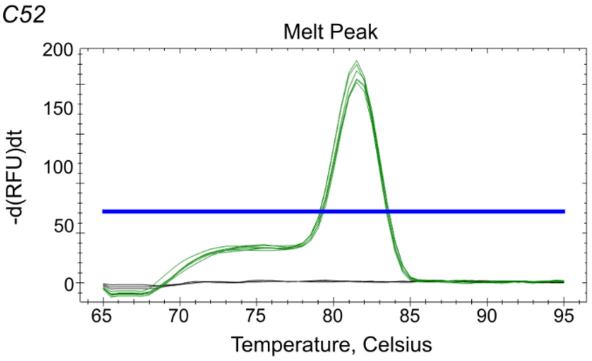

YLS8

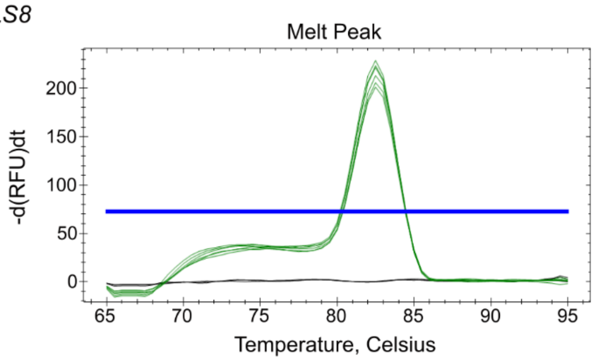

SAMDC

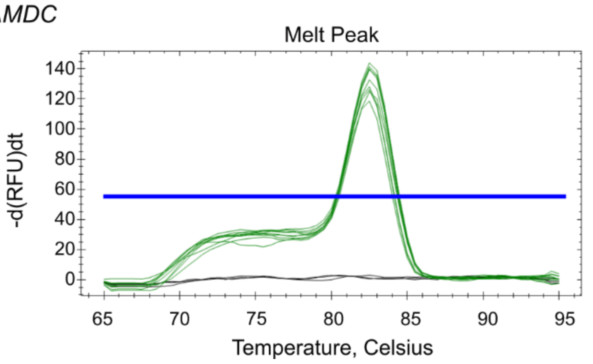

HIS3.3

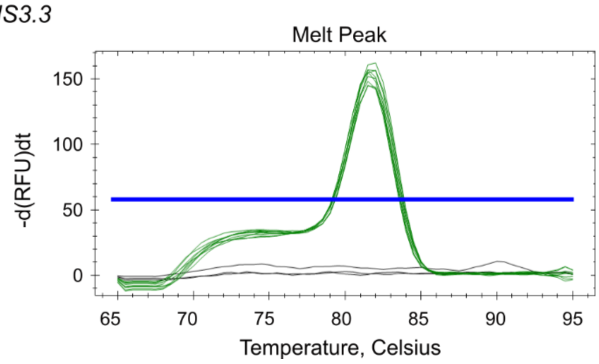

Figure S2. Melt curves of ten candidate reference genes (*UBC9*, *ACT7*, *GAPC-2*, *RCE1*, *PP2AA3*, *TUA2*, *SAC52*, *YLS8*, *SAMDC* and *HIS3.3*) showing the specificity of qPCR amplification. Green lines are the melt curves of each sample tested, black lines are the melt curves of NTCs and the horizontal blue lines represent the baseline thresholds.

Table S1. Expression values on EVOREPRO (https://evorepro.sbs.ntu.edu.sg/) [33]. Expression values are presented as transcripts per million (TPM) on four different types of tissues defined by [33]: male (microspore, bicellular pollen, tricellular pollen, mature pollen, pollen tube, generative cell and sperm cell), female (ovary, ovule and egg cell), flower (carpels, stigmatic tissue, stamen filaments, anthers, petals, sepals, flower buds and receptacles) and seeds (endosperm, young seed, seed and germinating seed).

**Gene Symbol**	**EVOREPRO - TPM**			
	**Male**	**Female**	**Flower**	**Seed**
UBC9	117.82	229.75	225.95	109.95
ACT7	60.75	467.27	595.82	326.93
GAPC2	397.10	353.65	565.74	344.34
RCE1	61.63	91.96	114.17	81.26
YLS8	112.85	428.76	266.24	326.73
TUA2	329.40	364.31	306.89	288.50
PP2AA3	31.23	55.18	36.57	36.31
SAC52	680.44	882.82	820.79	1072.44
SAMDC	839.40	707.57	741.30	477.41
HIS3.3	101.10	545.44	471.98	397.63

Table S2. List of target genes and primer sequences used to validate the reference genes.

**Locus**	**Gene Symbol**	**Gene Name**	**Primer Sequences Forward and Reverse (5' - 3')**	**Amplicon length (bp)**
AT5G14380	*AGP6*	*Arabinogalactan protein 6*	TTCTAAGTTAAGTCGTCCAC<br>GACATTTAGGTTTATATTTACTCC	135
AT3G01700	*AGP11*	*Arabinogalactan protein 11*	CCACGACTAATGTGAAGC<br>CAACAGGGGATGATGCTTTC	206
AT5G10430	*JAGGER*	*Arabinogalactan protein 4*	TCGCCACTTCAGCACTCGCTC<br>CGGGAGCACTGCTTGGGCTC	261

Table S3. List of the most stable reference genes for each set and subset of samples according to geNorm, NormFinder, BestKeeper, ΔCt method and RefFinder.

Most Stable								
	**Flower development**	**Flower (st 1-16)**	**Silique (st 17)**	**Pistil (st 12)**	**Anther (st 12)**	**Genotype**	**wt Col-0 vs. *jagger***	**wt No-0 vs. *agp6agp11***
**geNorm**	*TUA2* / *RCE1*	*YLS8* / *PP2AA3*	*TUA2* / *YLS8* / *PP2AA3* / *SAC52* / *HIS3.3* /	*TUA2* / *RCE1* / *YLS8* / *HIS3.3*	*TUA2* / *RCE1* / *UBC9* / *YLS8* / *SAC52* /	*HIS3.3* / *ACT7*	*HIS3.3* / *SAMDC*	*HIS3.3* / *YLS8* / *GAPC-2*
**NormFinder**	*RCE1*	*YLS8*	*SAC52* / *YLS8* / *TUA2* / *ACT7* / *HIS3.3* /	*RCE1* / *SAC52* / *YLS8* / *TUA2* / *HIS3.3* /	*RCE1* / *SAC52* / *YLS8* / *TUA2* / *ACT7* /	*YLS8*	*HIS3.3* / *SAMDC*	*HIS3.3* / *YLS8* / *GAPC-2*
**BestKeeper**	*UBC9*	*GAPC-2*	*UBC9* / *RCE1*	*UBC9*	*UBC9* / *RCE1* / *TUA2* / *SAC52* / *YLS8* /	*UBC9*	*UBC9*	*YLS8* / *GAPC-2* / *HIS3.3*
**ΔCt method**	*RCE1*	*YLS8*	*YLS8* / *SAC52* / *TUA2* / *HIS3.3* /	*YLS8* / *SAC52* / *TUA2* / *HIS3.3* /	*YLS8* / *SAC52* / *TUA2* / *RCE1* /	*YLS8*	*HIS3.3* / *SAMDC*	*YLS8* / *GAPC-2* / *HIS3.3*
**RefFinder**	*RCE1*	*YLS8*	*TUA2*	*RCE1*	*UBC9*	*YLS8*	*HIS3.3*	*YLS8*
